# Comparative Analysis of Classic Semen Extenders for Frozen–Thawed Boar Semen

**DOI:** 10.3390/ani15131885

**Published:** 2025-06-26

**Authors:** Yuting Kong, Mengqian He, Jun Gao, Jiehuan Xu, Naisheng Lu, Caifeng Wu, Lingwei Sun, Jianjun Dai

**Affiliations:** 1 College of Fisheries and Life Science, Shanghai Ocean University, Shanghai 201306, China; kytdyx2022@163.com; 2 Shanghai Municipal Key Laboratory of Agri-Genetics and Breeding, Institute of Animal Science and Veterinary Medicine, Shanghai Academy of Agricultural Sciences, Shanghai 201106, China; he1037247863@163.com (M.H.); gaojun@saas.sh.cn (J.G.); jiehuanxu810@163.com (J.X.); lunaisheng@saas.sh.cn (N.L.); wucaifengwcf@163.com (C.W.); 3 Key Laboratory of Livestock and Poultry Resources (Pig) Evaluation and Utilization, Ministry of Agriculture and Rural Affairs, Shanghai 201106, China; 4 Shanghai Engineering Research Center of Breeding Pig, Shanghai 201302, China

**Keywords:** boar semen, cryopreservation, semen extender, energy metabolism, spermatozoa motility

## Abstract

The physiological functions of mammalian sperm, such as motility, hyperactivation, and capacitation, require substantial energy. This study investigates the effects of two classic cryopreservation extenders—TCG (tris-citrate-glucose) and LEY (lactose-egg yolk)—on the energy metabolism of frozen–thawed boar semen. By comparing the quality indicators, key metabolite levels, and the activities of critical enzymes involved in glycolysis and the tricarboxylic acid cycle, we aim to understand how these different semen extenders influence the spermatozoa vitality of frozen–thawed boar semen. Following thawing, the LEY-cryopreserved sperm demonstrated significantly elevated motility parameters (viability, VCL, VSL, and VAP) and enhanced plasma membrane and acrosomal integrity compared with the TCG group (*p* < 0.05), though both cryopreserved groups exhibited significantly reduced performance relative to fresh semen controls. Cryopreservation markedly reduced intracellular adenosine triphosphate (ATP), pyruvate, and acetyl coenzyme A (A-CoA) levels (fresh > LEY > TCG; *p* < 0.05). The LEY-preserved spermatozoa retained higher activities of glycolysis-related enzymes (phosphofructokinase, PFK; pyruvate kinase, PK) compared with the TCG group, which, in turn, showed elevated lactate dehydrogenase (LDH) activity. Critically, TCG-suppressed pyruvate dehydrogenase (PDH) activity (*p* < 0.05) coincided with diminished A-CoA, indicating impaired mitochondrial oxidative phosphorylation. These results demonstrate LEY’s superior preservation of motility and membrane stability but highlight cryodamage-induced energy metabolism dysregulation, particularly TCG’s disruption of the glycolysis–TCA cycle coordination essential for spermatozoa function. In conclusion, the choice of semen extender has a significant impact on the energy metabolism and overall quality of frozen–thawed semen, highlighting the importance of optimizing cryopreservation protocols for improved spermatozoa viability and functionality.

## Simple Summary

The choice of semen extender is crucial for the in vitro preservation of mammalian sperm. This study compares two classic semen extenders for frozen–thawed boar semen—TCG (tris-citrate-glucose) and LEY (lactose-egg yolk)—to evaluate their effects on spermatozoa motility, structural integrity, and energy metabolism after thawing. The results indicate that the LEY-based semen extender significantly outperforms the TCG-based extender in maintaining the quality indicators of thawed semen, providing important insights for optimizing the cryopreservation techniques of boar semen.

## 1. Introduction

Boar semen cryopreservation relies on optimized freezing extenders to minimize freeze–thaw damage during freezing before immersion in liquid nitrogen (usually −196 °C) [1]. This cryogenic environment can effectively inhibit the physiological and metabolic activities of spermatozoa, thus achieving their long-term preservation. However, the commercial application of frozen boar spermatozoa remains limited, with only 1% of artificial inseminations worldwide utilizing frozen–thawed doses of boar semen, primarily due to declines in fertility and litter size after freezing [1]. Compared to fresh semen, frozen/thawed ejaculates are more susceptible to morphological changes in spermatozoa, such as acrosome abnormalities. Additionally, these spermatozoa exhibit lower motility, diminished mitochondrial potential, increased plasma membrane permeability, and elevated lipid peroxidation levels [2]. The severity of spermatozoa damage is tied to the different compositions of cryopreservation media, such as the inclusion of various compounds in freezing and thawing extenders [3,4]. Therefore, elucidating cryopreservation-induced damage in porcine spermatozoa through functional and metabolic indicator assessments will provide crucial insights for enhancing semen quality, viability, and cryostorage protocol optimization.

In mammalian physiology, essential activities, such as spermatozoa motility performance, hyperactivation, and energy acquisition, depend on a large supply of energy [5]. This energy is mainly produced through glycolysis, a key metabolic pathway that plays an important role in energy acquisition in organisms. Critical enzymes such as hexokinase (HK) and pyruvate kinase (PK) play crucial roles in this process, working in concert to break down glucose into pyruvate, with the accompanying release of energy [6,7]. In addition, the tricarboxylic acid cycle (TCA cycle), as a prevalent metabolic pathway in aerobic organisms, not only provides energy to the organism but is also the final metabolic pathway for the three major nutrients, namely, sugars, lipids, and proteins, thus playing a linking and pivotal role [8].

Spermatozoa exhibit remarkable metabolic plasticity, which provides them with the ability to adapt to changes in metabolic substrates or microenvironments and potentially respond to these changes through metabolic reprogramming [9,10,11]. The selection of seminal extender components plays a pivotal role in the in vitro preservation of mammalian spermatozoa [12]. This study specifically compares the effects of two classic semen extenders (tris-citrate-glucose, TCG; lactose-egg yolk, LEY) on the energy metabolism of frozen–thawed boar semen. The results will assess the impact of these different extenders on the vitality and energy metabolism of spermatozoa following cryopreservation.

During animal semen cryopreservation, the combined effects of spermatozoa’s heightened sensitivity to cryothermic stress, the inherent cytotoxicity of the semen extender, and their osmotic imbalance frequently trigger oxidative cascades, resulting in structural and functional impairments of the cryopreserved spermatozoa [13,14]. To thoroughly investigate the regulatory mechanisms of energy metabolism following the freezing of spermatozoa, this study examined conventional quality indicators of frozen spermatozoa, including the concentrations of key metabolites, such as glucose, lactose, and ATP, as well as the activities of critical enzymes involved in glycolysis and the tricarboxylic acid cycle, such as hexokinase (HK), pyruvate kinase (PK), and phosphofructokinase (PFK). By comparing the effects of two different semen extenders (TCG and LEY) on the energy metabolism of frozen semen, this study aims to improve the quality of frozen semen and promote the implementation of artificial insemination in pig farms. The results of this study provide a theoretical basis for improving the quality of pig semen cryopreservation and utilizing the advantages of effective breeding practices.

## 2. Materials and Methods

### 2.1. Reagents and Media

Unless otherwise noted, all reagents and chemicals were obtained from Sigma Chemical Co. (St. Louis, MO, USA). All reagents utilized were of analytical reagent grade. Ultrapure water (18.2 MΩ·cm; ELGA Purelab Classic, London, UK) was employed for solution preparation.

### 2.2. Animals

Eight Meishan breeder pigs (boars aged 2.0–3.0 years and weighing around 230 kg) were selected from Meishan Pig Farm in Jiading, Shanghai, China. All boars were used for breeding purposes at the experimental farm, were in good health, and had previously sired offspring. Throughout the experimental period, the boars were kept in separate pens and provided with the same feeding regimen and ad libitum access to autoclaved drinking water on a daily basis. All boars were fed a standard corn-/soy-based diet (3.15 ME/kg; 16% crude protein; 0.85% lysine; 0.75% calcium; and 0.65% phosphorus) twice daily. Boar health was monitored throughout the experimental period, and none of the animals had disease symptoms during the course of the trial. Semen samples were collected manually twice a week using the gloved-hand method over a period of three weeks and analyzed to ensure the quality and homogeneity of the ejaculates. A total of 48 ejaculates were manually collected. The semen samples had a simple milky white color, with no aversive odor or contamination. Following collection, semen samples were immediately wrapped in sterile gauze (≥4 layers) and placed in a portable incubator maintained at 37 ± 0.5 °C to prevent thermal fluctuation during transport. All samples were delivered to the laboratory within 2 h post-collection to preserve sperm metabolic homeostasis. All samples met the specified criteria, namely, a volume exceeding 200 mL, a concentration greater than 2 × 10^8^ spermatozoa/mL, and a motility above 80%, and they were subsequently utilized in further experiments [15].

### 2.3. Semen Cryopreservation

The semen samples were instantly equilibrated in a 17 °C refrigerator for 1 h and then centrifuged at 17 °C and 800× *g* for 15 min. The supernatant was discarded. Fresh ejaculates from eight Meishan boars (biological replicates) were collected across six independent collection rounds at weekly intervals. For each round, ejaculates were pooled and homogenized to minimize individual variation. The pooled semen samples were equally divided into two groups and diluted to 1.5 × 10^9^ spermatozoa/mL (using a Makler counting chamber; Sefi-Medical Instruments, Haifa, Israel) with the following two cryopreservation extenders: TCG and LEY. The TCG solution was prepared with an 80% mixture (100 mL diluted in double-distilled water) consisting of tris-hydroxymethyl aminomethane (2.42 g), citric acid (1.48 g), glucose (1.1 g), penicillin sodium (0.06 g), streptomycin sulfate (0.1 g), and 20% egg yolk [15]. The LEY solution was prepared with an 80% mixture (100 mL diluted in double-distilled water) consisting of 310 mM lactose (2.79 g), penicillin sodium (0.06 g), streptomycin sulfate (0.1 g), and 20% egg yolk [16]. The semen samples incorporated into each solution were incubated in a water bath maintained at 17 °C for 3 h. Subsequently, the semen was gradually cooled to 4 °C in a refrigerator and held for an additional 4 h to ensure equilibration. At 4 °C, an equal volume of pre-cooled solution II (solution I supplemented with an additional 3% glycerol) was added into each group. Following this, the samples were packaged into 0.5 mL labeled plastic straws (Minitüb Ibérica SL; Tarragona, Spain). The straws were then transferred to a programmable freezer (Icecube14S-B; Minitüb Ibérica, SL). The freezing protocol (IceCube 1810, SY-LAB; Neupurkersdorf, Austria) involved cooling at specified rates, as follows: 6 °C/min from 4 °C to −5 °C, 40 °C/min from −5 °C to −80 °C, maintenance at −80 °C for 30 s, and cooling at a rate of 60 °C/min from −80 °C to −150 °C [17]. The frozen samples were stored at −196 °C in liquid nitrogen for a duration of 12 weeks. After thawing in a 37 °C water bath, the cryopreserved semen samples underwent quality assessment within 30 min [18]. Each treatment was tested in triplicate to evaluate the effects of TCG and LEY on the energy metabolism of frozen–thawed boar semen.

### 2.4. Computer-Assisted Semen Analysis (CASA)

A computer-assisted semen analyzer (CASA, Version 12.3, Hamilton Thorne, MA, USA) and a microscope (Model BX51, Olympus, Tokyo, Japan) with a heating stage (37 °C) were used to evaluate the spermatozoa in terms of the straight-line velocity (VSL), average path velocity (VAP), and curvilinear velocity (VCL). The parameters for the analysis configuration were modified to the following threshold values: total frames: 45; frame rate: 60 Hz; minimum contrast: 46; minimum cell size: 7 pixels; alternate minimum contrast: 30; alternate cell size: 7 pixels; cell intensity: 50; range of static head size: 0.80–4.93; range of static head intensity: 0.49–1.68; and static elongation range: 22–84 [19]. A volume of 10 µL of the semen sample was placed on a Makler counting chamber (Makler^®^, 10 µm, Sefi Medical Instruments, Haifa, Israel), which had been preheated to 37 °C, and 4 microscopic fields per subsample were analyzed to include at least 1000 spermatozoa.

### 2.5. Plasma Membrane Integrity of Spermatozoa

The plasma membrane integrity of the spermatozoa was evaluated using a double-staining technique with Helixyte^®^ 7 (Aurogene, Rome, Italy) and propidium iodide (PI, Thermo Fisher Scientific, Waltham, MA, USA). Semen samples were diluted to a suitable concentration (1 × 10^6^ spermatozoa/mL) using phosphate-buffered saline (PBS) buffer and placed in a centrifuge tube. An appropriate volume of Helixyte^®^ 7 (5 µL) was added, followed by gentle mixing and incubation for 5 min to facilitate dye uptake. Afterward, PI was added at a concentration of 4 µg/mL, mixed gently, and incubated for an additional 5 min. The samples were then washed with PBS buffer to eliminate any unbound dye. A drop of the mixture was examined under a fluorescence microscope at 400× magnification, and more than 400 spermatozoa were randomly photographed. The green fluorescence emitted by Helixyte^®^ 7 indicates the presence of viable spermatozoa; conversely, the red fluorescence from PI signifies the presence of non-viable spermatozoa, thus indicating cell death. Plasma membrane integrity rate = (number of green fluorescent spermatozoa in the head/total number of spermatozoa) × 100%.

### 2.6. Acrosome Integrity of Spermatozoa

Fluorescein isothiocyanate-peanut agglutinin (FITC-PNA, Sigma-Aldrich, St. Louis, MO, USA) staining was employed to assess the acrosome integrity of the spermatozoa. In brief, an equal volume of the FITC-PNA staining solution was added to 100 μL of spermatozoa and gently mixed to ensure even distribution of the dye. The mixture was then incubated in a biochemical incubator at 37 °C for 30 min. Following incubation, a drop of the stained mixture was carefully aspirated onto a glass microscope slide to create a thin smear. The smear was air-dried at room temperature. Observations were conducted under a fluorescence microscope at 400× magnification, where more than 400 spermatozoa were randomly captured in images for analysis. Spermatozoa exhibiting bright green fluorescence indicate intact acrosomes; conversely, spermatozoa that do not fluoresce or show weak fluorescence are indicative of compromised acrosomes. Acrosome integrity rate = (number of green fluorescent spermatozoa with intact acrosome/total spermatozoa) × 100%.

### 2.7. Determination of Key Enzyme Activities

The activities of various glycolysis and TCA enzymes in the semen samples were assessed using specific assay kits from the Nanjing Jiancheng Institute of Bioengineering (Nanjing Jiancheng, Nanjing, China), including an HK assay kit (No. A077), PK assay kit (No. A076), PFK assay kit (No. A129), lactate dehydrogenase (LDH) assay kit (No. A020), citrate synthase (CS) assay kit (No. A108), isocitrate dehydrogenase (IDH) assay kit (No. H311), pyruvate dehydrogenase (PDH) assay kit (H262), and acetyl coenzyme A (acetyl-CoA) assay kit (No. H232). For enzyme activity detection, the semen samples were collected and diluted to an appropriate concentration (1 × 10^5^ spermatozoa/mL) using the provided assay buffer. The assay reagents and substrates were prepared according to the instructions provided in each kit. In a 96-well microplate, the diluted semen sample (10 µL) was added to designated wells, followed by the addition of the reaction buffer and specific substrates. The plate was incubated at the recommended temperatures, namely, 25 °C for HK, PK, PFK, and LDH and 37 °C for CS, IDH, and PDH, for the specified duration (15–60 min) to allow for enzyme-catalyzed reactions. After incubation, a stopping solution was added, and the absorbance or fluorescence of the reaction mixtures was measured using a microplate reader (Synergy H1 microplate reader, BioTek, Winooski, VT, USA) at the following specified wavelengths: 340 nm for HK, PK, PFK, LDH, IDH, PDH, and acetyl-CoA and 412 nm for CS. Enzyme activities were calculated based on the readings compared to standard curves generated from known concentrations of the reaction products, ensuring accurate and reliable measurements of the enzyme activities in the semen samples.

The ATP levels in the semen samples were assessed using a luminescence-based ATP assay kit (Roche, ATP Bioluminescence Assay Kit HS II), as previously described [20]. Initially, 100 µL of the diluted semen sample suspension was combined with 100 µL of Cell Lysis Reagent, followed by vortex-mixing (2000 rpm, 30 s) and equilibration at 25 °C ± 1 °C for 5 min. The resultant cell lysate was then subjected to centrifugation at 12,000× *g* for 2 min. The supernatant was carefully collected and subsequently frozen in liquid nitrogen. Bioluminescence was quantified in triplicate using a luminometer (BioTeK Synergy HT, Winooski, VT, USA) in 96-well plates. Following automated injection of 50 μL luciferase working solution (containing 0.5 mM D-luciferin, 1 mM ATP, and 20 mM Tris-acetate buffer, pH 7.8) into a 50 μL sample aliquot, bioluminescence signals were acquired in kinetic mode (1 s latency for mixing stabilization) with 10 s continuous photon counting at 25 °C. Standard curves were established with solutions containing known concentrations of ATP, which were diluted in mTALP and mixed with Cell Lysis Reagent in proportions that matched those of the samples.

Pyruvate concentrations were determined fluorometrically (Pyruvate Assay Kit, BioVision, Cat# K609) based on NADH-coupled enzymatic oxidation. In summary, 50 µL of each sample was placed in duplicate into 96-well plates, along with pyruvate standards. Subsequently, a 50 μL reaction mixture was added to each sample and standard, which consisted of 47.6 µL pyruvate assay buffer, 0.4 µL pyruvate probes, and 2 µL of enzyme mix. After incubating at room temperature for 30 min, the fluorescence was measured at excitation/emission wavelengths of 535/590 nm. The pyruvate concentrations in the samples were determined using a standard curve and reported as nmol/mg protein.

### 2.8. Statistical Analysis

A one-way analysis of variance (ANOVA) was performed using SPSS 27.0 (SPSS Inc., Chicago, IL, USA) software, and multiple comparisons were conducted using the LSD method. Prior to the ANOVA, normality tests, such as the Shapiro–Wilk test, were conducted to assess whether the data followed a normal distribution. If the data met the assumption of normality, the ANOVA was applied. If the normality assumption was violated, a non-parametric alternative, such as the Kruskal–Wallis test, was used instead. The results are expressed as the mean ± standard error mean (SEM) and were plotted using GraphPad Prism 9.5 (GraphPad Software Inc., CA, USA). A *p*-value of <0.05 indicates a significant difference, while a *p*-value of >0.05 indicates a non-significant difference.

## 3. Results

### 3.1. Effect of Different Semen Extenders on the Motility Parameters of Boar Spermatozoa in Cryopreserved Ejaculates

As shown in Table 1, fresh semen demonstrated significantly higher motility parameters than cryopreserved semen (*p* < 0.05). Among the frozen semen groups, the LEY group demonstrated significantly higher viability, motility, and motility parameters (including VCL, VSL, and VAP) compared to the TCG group (*p* < 0.05). Additionally, the LEY group outperformed the TCG group in terms of all assessed motility parameters. Video S1 displays videos of the three groups of semen samples.

### 3.2. Effect of Different Semen Extenders on Plasma Membrane and Acrosome Integrities of Boar Spermatozoa in Cryopreserved Ejaculates

**Figure 1:**
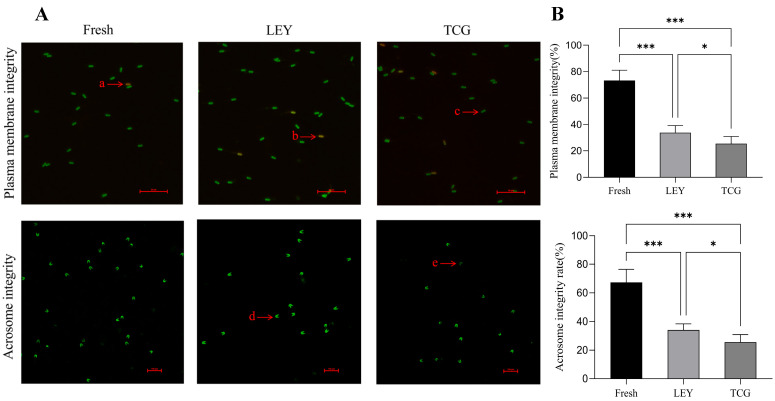
Plasma membrane integrity and acrosome integrity of fresh and frozen boar spermatozoa (*n* = 8 boars and *n* = 48 ejaculates). (**A**) Fluorescent staining of spermatozoa plasma membranes and acrosomes. In the assessment of plasma membrane integrity, a and b indicate broken plasma membranes, while c represents an intact plasma membrane. For acrosomal integrity, d denotes smooth and intact acrosomes without damage, and e indicates incomplete acrosomes. (**B**) Statistical analysis of plasma membrane integrity and acrosomal integrity rates of spermatozoa. Values are the mean ± SEM. * *p* < 0.05, *** *p* < 0.001.

As shown in Figure 1B, the fresh semen group displayed significantly higher plasma membrane and acrosomal integrities than both the LEY and TCG cryopreserved groups (*p* < 0.05). Notably, the LEY group displayed higher plasma membrane and acrosomal integrity retention compared with the TCG group, with statistically significant differences observed (*p* < 0.05).

### 3.3. Effect of Different Semen Extenders on the Content of Key Energy Metabolites in Boar Spermatozoa in Cryopreserved Ejaculates

**Figure 2:**
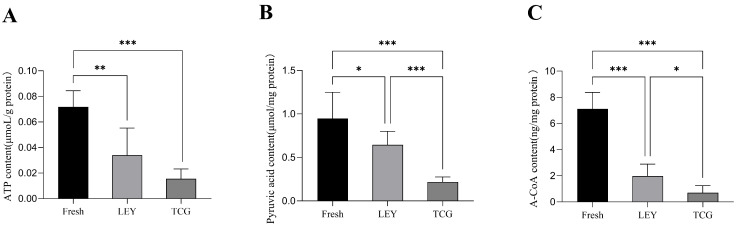
Statistical analysis of spermatozoa ATP (**A**), pyruvate (**B**), and acetyl coenzyme A (A-CoA; (**C**)) levels (*n* = 8 boars and *n* = 48 ejaculates). Values are the mean ± SEM. * *p* < 0.05, ** *p* < 0.01, *** *p* < 0.001.

As shown in Figure 2A, the ATP level in the fresh semen group was significantly higher than that in the LEY and TCG groups. Figure 2B indicates that the pyruvate content in the fresh semen group was likewise significantly higher than those in the LEY and TCG groups. Figure 2C demonstrates that the acetyl-CoA content was significantly lower in both the LEY and TCG groups compared with the fresh semen group. Additionally, Figure 2 demonstrates the trends in ATP, pyruvate, and acetyl-CoA levels in the three groups, in decreasing order, as follows: fresh group > LEY group > TCG group.

### 3.4. Effect of Different Semen Extenders on the Activities of Key Glycolysis Enzymes in Boar Spermatozoa in Cryopreserved Ejaculates

**Figure 3:**
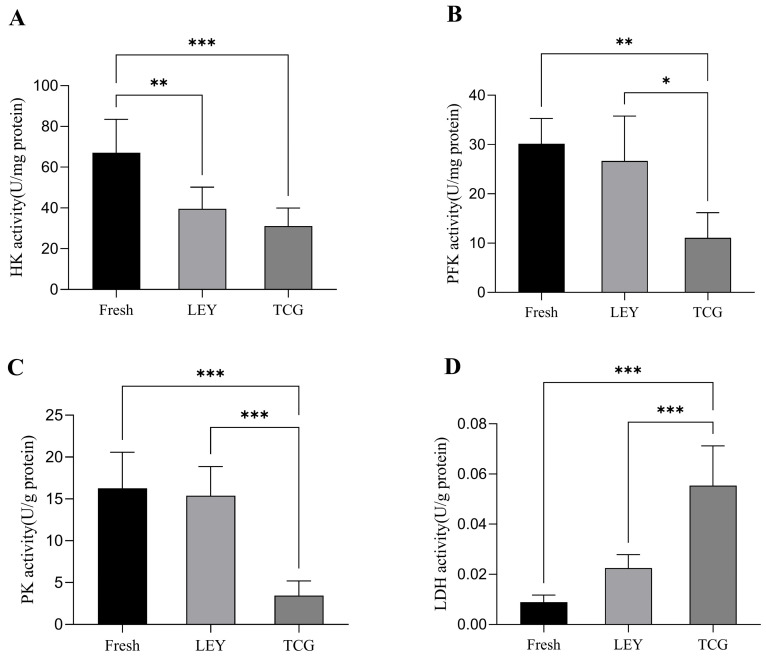
Statistical analysis of key glycolysis enzyme activities in spermatozoa (*n* = 8 boars and *n* = 48 ejaculates): (**A**) hexokinase (HK) enzyme activity; (**B**) phosphofructokinase (PFK) enzyme activity; (**C**) pyruvate kinase (PK) enzyme activity; (**D**) lactate dehydrogenase (LDH) enzyme activity. Values are the mean ± SEM. * *p* < 0.05, ** *p* < 0.01, *** *p* < 0.001.

As shown in Figure 3A, HK enzyme activity was significantly higher in the fresh semen group than in the LEY and TCG groups. Figure 3B indicates that PFK enzyme activity was also higher in the fresh semen group compared with the frozen groups, with the LEY group exhibiting significantly higher PFK activity than the TCG group. Figure 3C demonstrates that PK activity in both the fresh semen and LEY groups was significantly higher than that in the TCG group. Finally, as shown in Figure 3D, LDH enzyme activity was significantly higher in the TCG group compared with the fresh semen and LEY groups.

### 3.5. Effect of Different Semen Extenders on the Activities of Key Enzymes of the Tricarboxylic Acid Cycle in Boar Spermatozoa in Cryopreserved Ejaculates

**Figure 4:**
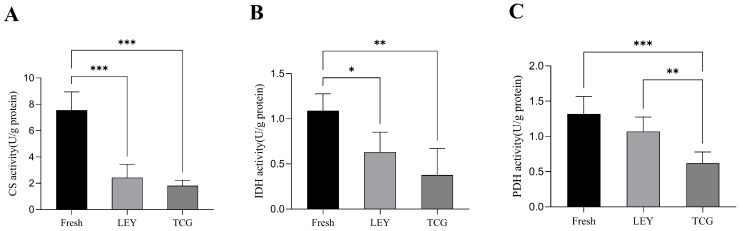
Statistical analysis of key activities of tricarboxylic acid cycle enzymes in spermatozoa (*n* = 8 boars and *n* = 48 ejaculates): (**A**) citrate synthase (CS) enzyme activity; (**B**) isocitrate dehydrogenase (IDH) enzyme activity; (**C**) pyruvate dehydrogenase (PDH) enzyme activity. Values are the mean ± SEM. * *p* < 0.05, ** *p* < 0.01, *** *p* < 0.001.

As shown in Figure 4A, CS enzyme activity was significantly higher in the fresh semen group than in the LEY and TCG groups. Figure 4B indicates that IDH enzyme activity was also significantly higher in the fresh semen group compared with the LEY and TCG groups. Additionally, Figure 4C indicates that PDH enzyme activity in the TCG group was significantly lower than that in the fresh semen and LEY groups.

## 4. Discussion

The main objective of this study was to investigate the cryoprotective efficacy of LEY versus TCG semen extenders on the motility, plasma membrane integrity, acrosome integrity, and energy metabolism of boar spermatozoa in cryopreserved ejaculates. We compared experimental data from the following three treatment groups: a fresh semen group, a TCG group, and an LEY group. The analysis aimed to reveal how these semen extenders influence the physiological function and quality of semen during the cryopreservation process.

Spermatozoa motility and viability are critical parameters for assessing male fertility [21]. Spermatozoa motility refers to the effective movement of sperm, particularly progressive motility, which is essential for reaching the egg. In contrast, spermatozoa viability measures the proportion of live spermatozoa in a sample, which is vital for successful fertilization. High motility and viability are necessary for spermatozoa to navigate the female reproductive tract and achieve fertilization [22]. When spermatozoa undergo in vitro cryopreservation, the plasma membrane structure of these cells can be compromised due to the rapid decrease in temperature. This rapid cooling process can lead to the formation of ice crystals within the cells, which can physically damage the spermatozoa and reduce their motility and viability. Furthermore, the hypertonic conditions that arise during the freezing process can result in protein degradation and phosphorylation within the spermatozoa [23]. Proteins essential for spermatozoa function may unfold and lose their biological activity, impairing the sperm’s ability to fertilize an egg [24]. Additionally, the formation of ice crystals can inflict irreversible mechanical damage upon the spermatozoa [25]. These ice crystals can pierce through the cell membranes and organelles, causing severe structural damage that cannot be repaired.

Our data demonstrate that spermatozoa from ejaculates cryopreserved with the LEY extender maintained significantly higher motility and viability than the TCG extender, suggesting that LEY cryoprotectant formulation may enhance metabolic stability during cryopreservation. This outcome indicates that the LEY extender may provide a more protective environment for the semen during the freezing and thawing process, potentially due to its unique composition, which helps stabilize cell membranes and proteins.

The plasma membrane integrity of spermatozoa is a critical parameter in evaluating semen quality and fertility potential [26]. It refers to the proportion of spermatozoa whose membrane structure remains intact in a semen sample. A healthy plasma membrane is directly correlated with the ability of the spermatozoa to undergo capacitation, a process vital for achieving fertilizing competence [27]. Acrosome integrity is also essential for the successful participation of semen in fertilization [28]. Our study demonstrated that spermatozoa from ejaculates cryopreserved using the LEY extender retained significantly greater structural integrity—including the plasma membrane and acrosomal compartments—compared to those cryopreserved with the TCG extender, likely associated with improved metabolic substrate utilization provided by the extender’s composition during cryopreservation. This result further verifies the significant advantage of the LEY extender in protecting the structural integrity of spermatozoa within cryopreserved ejaculates, which may provide a scientific basis for improving semen quality and potentially enhancing the success rates of assisted reproductive techniques.

Spermatozoa possess a unique ability to self-regulate and can independently adjust their metabolic processes according to their energy requirements [29]. A key role in this process is played by lactate dehydrogenase, which catalyzes the conversion of pyruvate to lactate, a reaction essential for sustained ATP production during glycolysis [30]. The experimental results of the present study reveal that, among the cryopreserved semen samples, ATP, pyruvate, and acetyl-CoA levels were significantly higher in the LEY group than in the TCG group. The observed improvement in energy metabolism is in line with previous findings showing that coenzyme Q10 supplementation enhances sperm viability, plasma membrane integrity, and antioxidant capacity [31].

Glycolysis and oxidative phosphorylation are the two primary pathways responsible for ATP production in spermatozoa [32]. These metabolic processes are crucial for generating the energy required for spermatozoa motility and functionality. Research indicates that ATP generated by mitochondria via oxidative phosphorylation might not be adequate to sustain spermatozoa motility, suggesting that the glycolytic pathway could be the preferred energy source for spermatozoa during movement [32]. In our current experiment, we noted that the activities of several key enzymes, including HK, PK, and PFK, were more pronounced in the LEY group than in the TCG group. This finding suggests that the overall glycolytic activity of the LEY group was likely higher than that of the TCG group. An increased activity of these enzymes suggests a higher rate of glycolysis in the LEY group, indicative of a more efficient energy production mechanism for sustaining spermatozoa motility. Furthermore, we discovered that LDH activity was notably higher in the TCG group than in the LEY group. This observation may suggest increased conversion of pyruvate to lactate in the TCG group. Consequently, we hypothesized that pyruvate produced through glycolysis is more frequently converted into lactate in the TCG group, potentially as a compensatory mechanism to manage energy demands under the stress of the freezing process.

The TCA cycle is a fundamental metabolic pathway that plays a crucial role in cellular energy metabolism [33]. It is responsible for the oxidation of various nutrients, such as carbohydrates, amino acids, and fatty acids, which can be converted into intermediates of the TCA cycle through a series of metabolic pathways [34]. The TCA cycle is tightly regulated to ensure that the energy needs of the cell are met efficiently and effectively. The regulation of TCA cycle enzymes is influenced by various factors, including the availability of substrates, the energy status of the cell, and the presence of hormones and other signaling molecules [35]. In the context of spermatozoa, the proper functioning of the TCA cycle is essential for maintaining motility and viability, which are critical for successful fertilization [36]. In this study, the enzyme activities of CS, IDH, and PDH were found to be at their highest levels in the fresh semen group, followed by the LEY group, which exhibited notably higher enzyme activities than the TCG group. These findings suggest that the freezing treatment has a significant impact on the activities of key enzymes involved in the spermatozoa TCA cycle, leading to a decrease in their activity. Both CS and PDH enzymes are pivotal in the production of A-CoA, a critical molecule in cellular metabolism [37]. The enzyme activities of CS and PDH were observed to be higher in the LEY group than in the TCG group. Consequently, the A-CoA content in the LEY group was also significantly higher than that in the TCG group. This observation is in line with the previously described trends in the changes in the contents of key metabolites, further underscoring the importance of the TCA cycle in maintaining the metabolic integrity of spermatozoa [35].

The LEY group demonstrated superior sperm quality indicators—including motility, plasma membrane integrity, and acrosome integrity—compared with the TCG group. This advantage may be associated with improved metabolic substrate utilization, as the spermatozoa in the LEY group may have more effectively utilized the components of the semen extender, thereby enhancing their metabolic processes for energy production. This advantage may be explained by more efficient metabolic substrate utilization, where spermatozoa in the LEY group effectively utilized semen extender components, possibly enhancing their metabolic processes for energy production. Additionally, LDH activity was significantly lower in the LEY group compared with the TCG group, while PDH activity was significantly higher. This suggests that pyruvate in the LEY group may have been preferentially converted to A-CoA for entry into the TCA cycle instead of being converted to lactate. Prior research indicates that cryopreservation alters protein expression and acetylation in spermatozoa, which may regulate pyruvate metabolism through signaling pathways [38]. The higher activity of the TCA cycle enzymes in the LEY group may have resulted in greater ATP production, which may have contributed to enhanced spermatozoa motility and quality. In contrast, in the TCG group, the observed higher LDH activity combined with lower PDH activity suggests that a greater proportion of pyruvate may have been converted into lactate, with only a smaller fraction entering the TCA cycle via conversion to A-CoA. This limited A-CoA supply may have contributed to lower TCA cycle enzyme activity and reduced ATP production in the TCG group, ultimately resulting in lower motility and quality parameters compared with the LEY group.

## 5. Conclusions

This study further confirms the significant advantages of the LEY extender over TCG in the cryopreservation of boar sperm. Our findings indicate that the LEY extender demonstrated superior cryoprotective efficacy relative to the TCG formulation in preserving spermatozoa motility, cellular ultrastructural integrity, and energy metabolic homeostasis within cryopreserved ejaculates. These findings not only provide a solid theoretical basis for optimizing porcine-semen cryopreservation technology may offer detailed data support for practical applications. However, it is important to note that fertilization ability assessments through in vitro fertilization (IVF) were not performed due to technical constraints in our current experimental setup. The absence of IVF validation limits direct conclusions about the functional competence of cryopreserved semen in achieving successful embryogenesis. Future studies should prioritize integrating IVF trials to correlate post-thaw semen quality parameters (e.g., motility and DNA integrity) with actual fertilization outcomes. This may further validate the practical applicability of lactose-based cryopreservation protocols in assisted reproductive technologies.

## Figures and Tables

**Table 1:** Effect of different semen extenders on the motility of frozen–thawed boar semen (*n* = 8 boars and *n* = 48 ejaculates).

Variable (Unit)	Fresh Semen Group	LEY Group	TCG Group
Viability (%)	77.70 ± 3.92 ^a^	52.33 ± 2.57 ^b^	42.85 ± 3.00 ^c^
Motility (%)	57.86 ± 5.59 ^a^	42.12 ± 4.32 ^b^	35.19 ± 2.92 ^b^
VCL (µm/s)	70.94 ± 2.03 ^a^	58.62 ± 6.62 ^b^	49.64 ± 4.21 ^c^
VSL (µm/s)	52.46 ± 2.73 ^a^	30.09 ± 7.02 ^b^	21.66 ± 5.97 ^c^
VAP (µm/s)	56.89 ± 3.01 ^a^	43.77 ± 7.56 ^b^	39.28 ± 5.58 ^c^

VSL, straight-line velocity; VAP, average path velocity; VCL, curvilinear velocity. Values are expressed as the mean ± SEM, with different lowercase letters indicating significant differences (*p* < 0.05).

## Data Availability

The original contributions presented in this study are included in the article/Supplementary Material. Further inquiries can be directed to the corresponding authors.

## Supplementary Materials

The following supporting information can be downloaded at https://www.mdpi.com/article/10.3390/ani15131885/s1, Video S1: videos of three groups of semen samples.
